# Combined B-vitamin supplementation on homocysteine and vascular outcomes in coronary heart disease: a meta-analysis

**DOI:** 10.1080/07853890.2026.2622208

**Published:** 2026-01-30

**Authors:** Liping Guo, Xiangfen Shi, Gaobiao Wang, Wenchao Han, Rui Ding, Shihao Wang, Dongdong Yuan

**Affiliations:** a Department of Pharmacy, The 7th People’s Hospital of Zhengzhou, Zhengzhou, Henan Province, People’s Republic of China; b Department of Pharmacy, The First Affiliated Hospital of Zhengzhou University, Zhengzhou, Henan Province, People’s Republic of China; c Clinical Trial Institution for Drugs, The 7th People’s Hospital of Zhengzhou, Zhengzhou, Henan Province, People’s Republic of China; d School of Pharmaceutical Sciences, Zhengzhou University, Zhengzhou, Henan Province, People’s Republic of China

**Keywords:** Homocysteine, vitamin B, coronary heart disease, vascular stenosis, cardiovascular events

## Abstract

**Objective:**

Hyperhomocysteinemia (Hcy) independently predicts coronary heart disease (CHD) and adverse cardiovascular events. Although folic acid plays a key role in Hcy metabolism, the effect of combined B-vitamin supplementation (folic acid, VB6, and VB12) on clinical outcomes in CHD remains uncertain.

**Methods:**

A systematic search of PubMed, Embase, and the Cochrane Library was conducted from inception through April 2025 using MeSH terms including “folic acid,” “vitamin B6,” “vitamin B12,” “coronary heart disease,” and “homocysteine.” A random-effects model was used for meta-analysis.

**Results:**

Thirteen studies involving 14,539 participants were included in the meta-analysis (7,338 patients treated with folic acid combined with vitamin B complex and 7,301 controls). Combined B-vitamin supplementation significantly reduced serum Hcy levels [mean difference: −2.36; 95% confidence interval (CI): (−3.09 to −1.62); *p* < 0.01] compared with any single-nutrient regimen. The incidence of vascular restenosis was lower in the intervention group than in the control group (risk ratio: 0.65; 95% CI: 0.44–0.95; *p* < 0.05). However, no significant differences were observed in the incidence of major cardiovascular events (*p* = 0.78) or cardiovascular-related mortality (risk ratio: 0.96; 95% CI: 0.85–1.07; *p* = 0.44).

**Conclusion:**

Combined B-vitamin supplementation effectively lowers serum Hcy levels and the incidence of vascular restenosis in patients with CHD. However, its impact on cardiovascular events and mortality remains inconclusive.

## KEY MESSAGES

Meta-analysis of 13 RCTs (n = 14,539): combined B vitamins lower serum Hcy

Combined B-vitamin supplementation lowers vascular restenosis in CHD patients

Exerts no significant impact on major CV events or mortality

Hcy-lowering appears CHD-specific; broader CV benefits remain unproven

## Graphical abstract

**Figure:**
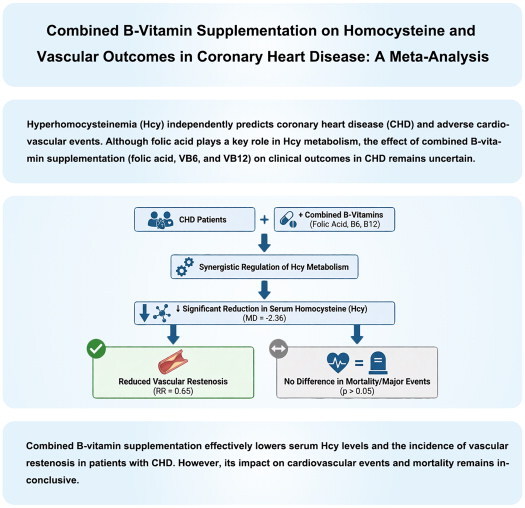

## Introduction

Coronary heart disease (CHD) is a major cardiovascular disorder threatening human health, and extensive research has indicated that elevated plasma homocysteine (Hcy) levels are recognized as an important independent risk factor for CHD development [1,2]. Impaired Hcy metabolism causes hyperhomocysteinemia, which promotes atherosclerosis by inducing endothelial injury, platelet aggregation, and vascular smooth muscle proliferation [3]. Consequently, reducing Hcy levels has become an important target in CHD prevention.

The limited efficacy of individual B-vitamin supplementation in certain health contexts has prompted further exploration into the role of vitamin B in the metabolism of Hcy [4]. This inquiry has revealed that the metabolic homeostasis of Hcy relies on two highly coordinated biochemical pathways, each intricately involving specific members of the vitamin B family. The first pathway is the remethylation pathway. In this process, Hcy is remethylated to methionine under the catalysis of the methyl group provided by the folic acid derivative 5-methyltetrahydrofolate in conjunction with the vitamin B12 (cobalamin)-dependent enzyme methionine synthase (MS). Vitamin B12 acts as an essential cofactor for MS, directly mediating the transfer of the methyl group. This remethylation process is crucial for maintaining the balance of Hcy levels in the body because it ensures that excess Hcy is efficiently converted back into methionine, a vital amino acid with numerous biological functions [5].

The second pathway is the sulfurization pathway. In this process, vitamin B6—particularly in its active form, pyridoxal-5′-phosphate (PLP)—acts as a coenzyme for the enzyme cystathionine β-synthase (CBS). CBS catalyzes the condensation of Hcy and serine to form cystathionine, which is subsequently converted into cysteine by γ-cystathionase [6]. Cysteine then serves as a building block for critical antioxidant molecules, including glutathione. This pathway plays a vital role in detoxifying Hcy and transforming it into metabolites that contribute to maintaining the body’s redox balance and overall metabolic function. In summary, the remethylation and transsulfuration pathways tightly regulate Hcy levels, with each pathway relying on specific B vitamins—folic acid, vitamin B12, and vitamin B6—as essential cofactors. The interplay among these vitamins underscores the importance of adequate and balanced B-vitamin intake to support Hcy metabolism. Consequently, combined supplementation with these B vitamins may offer metabolic and cardiovascular benefits by promoting Hcy clearance and supporting cellular health [7].

Folic acid acts as a methyl donor, vitamin B12 facilitates methyl group transfer, and vitamin B6 drives the transsulfuration pathway. Together, these three B vitamins regulate Hcy metabolism through complementary and interconnected mechanisms [8]. Relying on a single vitamin may be insufficient owing to metabolic limitations—for example, the “folic acid trap,” in which excess folate can mask vitamin B12 deficiency and impair Hcy clearance. In contrast, combined supplementation with folic acid, vitamin B6, and vitamin B12 is more likely to enhance metabolic efficiency and promote effective Hcy reduction [9]. We speculate that this combined approach may lower Hcy levels more effectively, thereby reducing the risk of CHD. This highlights the need for a comprehensive approach to manage Hcy metabolism, as the interplay between these vitamins is crucial for maintaining optimal Hcy levels and preventing cardiovascular complications [10].

Based on this rationale, we conducted a comprehensive analysis of the effects of combined B-vitamin supplementation (folic acid, B6, and B12) on Hcy levels and its preventive potential in CHD [11]. Our study aimed to determine whether multivitamin therapy offers greater Hcy-lowering benefits than single-vitamin approaches. We also examined whether elevated Hcy levels contribute similarly to other cardiovascular diseases, with implications for broader prevention strategies and clinical guidelines.

## Materials and methods

This meta-analysis followed the Preferred Reporting Items for Systematic Reviews and Meta-Analyses guidelines [12] and was registered under CRD420251109709.

### Search strategy

We systematically searched PubMed, Embase, and the Cochrane Library from January 2002 to April 2025 using the MeSH terms “folic acid,” “vitamin B6,” “vitamin B12,” “coronary heart disease,” and “homocysteine.” The search strategy was designed to identify all relevant studies published in English and other languages. Notably, prior to the initiation of this study, several key meta-analyses and reviews had explored the association between folate, homocysteine (Hcy), and coronary heart disease (CHD). Clarke and Lewington systematically reviewed clinical trials of folate for Hcy-related CHD and confirmed the availability of numerous relevant trials before 2002 [13]. Consistent with their findings, our meta-analysis also observed no significant causal relationship between folate supplementation-induced Hcy reduction and CHD risk reduction.

### Selection criteria

Studies were included if they met the following criteria: (1) randomized controlled trials (RCTs) involving human participants; (2) patients diagnosed with acute or chronic CHD, including myocardial infarction); (3) intervention group receiving folic acid in combination with B vitamins (B6, B12, or both), and a control group receiving sham treatment or a combination without folic acid; (4) clear description of participant selection, grouping, and control processes; (5) reported outcome indicators, including serum Hcy levels, incidence of vascular restenosis, cardiovascular events, or mortality. Studies were excluded if they were case reports, protocol studies, reviews, or conference abstracts, or if they lacked relevant outcome data.

### Exclusion criteria

The following types of studies were excluded: (1) case reports, protocol studies, reviews, or conference abstracts; (2) studies involving nonhuman subjects or participants without CHD; (3) studies lacking an intervention or control group; and (4) studies without relevant outcome indicators or sufficient data.

### Data extraction and quality assessment

Two independent authors conducted the literature screening, with disagreements resolved by the corresponding author. EndNote was used to manage and screen all studies. Initial screening involved reviewing titles and abstracts to identify studies that met predefined inclusion and exclusion criteria. Full texts of the selected studies were then assessed in detail against these criteria. The reviewers compared their screening outcomes, and any discrepancies were resolved through discussion. If consensus could not be reached, a third reviewer mediated the decision. The following data were independently extracted from each study: first author, year of publication, study location, participant characteristics, intervention details, sample size, and measured outcomes. The risk of bias for the 13 included RCTs was assessed using the Cochrane Risk of Bias 2.0 tool and is summarized in Table S1.

**The Cochrane Risk of Bias 2.0 tool was used to evaluate the quality of the included RCTs.** This standard includes five domains (randomization process, implementation bias, data bias, data measurement bias, and selection bias) and one overall bias assessment. Herein, “low risk,” “some concerns of risk,” and “high risk” were used to evaluate each domain (or overall bias).

### Data extraction and outcome measures

Given the large number of outcome measures reported in the literature, we prioritized the outcome measures that have been most frequently reported in the literature as the primary outcome measures. Based on this standard, the following primary outcome measures were collected in this study: (1) the expression level of total Hcy (TH) in the serum after intervention, continuous data, measured in μmol/L; (2) incidence of vascular restenosis: defined as the ratio obtained by dividing the number of patients with definite vascular stenosis who require vascular reconstruction by the number of participants in the group; (3) incidence of cardiovascular events: defined as the ratio obtained by dividing the number of patients who experienced any cardiovascular event (fatal or nonfatal myocardial infarction) by the number of participants in the group; (4) cardiovascular-related mortality: defined as the mortality rate caused by cardiovascular events divided by the number of participants in the group.

### Statistical analysis

All analyses were performed using R software. Effect sizes for continuous variables were expressed as combined mean differences (MD) with 95% confidence intervals (95% CIs), while those for discrete variables were expressed as combined risk ratios (RRs). Forest plots were used for visual presentation. Study heterogeneity was assessed using the I^2^ statistic and Cochrane’s Q test. An I^2^ value of <50% or *p* > 0.05 indicated no significant heterogeneity, allowing the use of a fixed-effects model [14]. In cases of heterogeneity, a random-effects model was used, with the combined values calculated using the Der Simonian and Laird methods. Sensitivity analyses were performed by sequentially removing outliers individually to assess their impact on the overall outcomes. Publication bias was assessed using the Begg’s and Egger’s tests and visualized through funnel plots [15,16]. Two-tailed *p-*values < 0.05 were used to denote statistical significance.

## Results

### Search results

**Figure 1.**
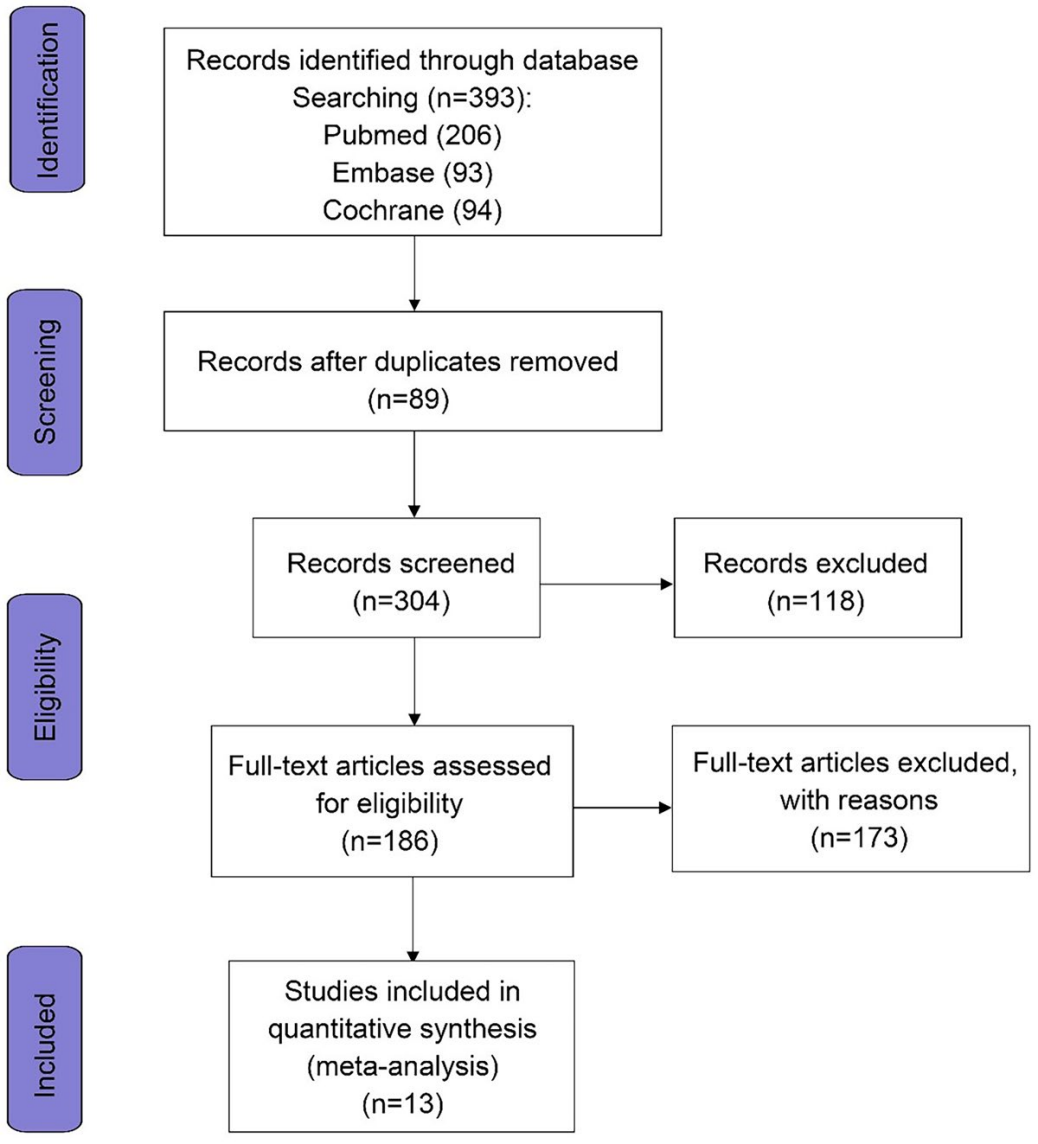
Flow chart of the study selection process.

Figure 1 presents a flowchart illustrating the systematic literature search strategy, delineating the step-by-step process of study identification. In cases of missing data, corresponding authors were contacted directly. From an initial pool of 393 records, 13 articles were deemed eligible for inclusion in the current meta-analysis based on the aforementioned criteria [17–29]. All included studies were RCTs, comprising 14,539 participants (7,338 patients treated with folic acid combined with vitamin B complex and 7,301 controls). In the intervention group, 11 articles adopted the combined treatment plan of folic acid, vitamin B6, and vitamin B12, whereas 2 articles adopted the combination of folic acid and vitamin B12. Table 1 presents a summary of the included studies.

**Table 1.** Main characteristics of the 13 included studies.

Data source	Primary disease	Total subjects	Age (years)	Male %	E/C	Intervention	FA Dose (/d)	Follow-up time	Outcome
Bønaa et al. 2006 [17]	AMI	1880	64 ± 12	73%	943 / 937	folic acid, B6, B12	0.8 mg	2 months	TH level, Incidence of major adverse event, Mortality
Chambers et al. 2000 [18]	CHD	89	57 ± 6	nr	59 / 30	folic acid, B12	5mg	2 months	TH level
Lobo et al. 1999 [19]	CHD	45	61 ± 11	77%	23 / 22	folic acid, B6, B12	5mg	3 months	TH level
Schnyder et al 2002 [20]	Patients undergo angioplasty	453	63 ± 11	79%	272 / 281	folic acid, B6, B12	1mg	6 months	TH level, Incidence of major adverse event, restenosis rate, Death rate from cardiovascular causes
Albert et al. 2008 [21]	CHD	5442	63 ± 9	NR	2721 / 2721	folic acid, B6, B12	2.5 mg	12 months	Incidence of major adverse event, Death rate from cardiovascular causes
Bleie et al. 2007 [22]	CHD	46	62(38–80)	77%	22 / 24	folic acid, B6, B12	5 mg	6 months	Inflammatory markers, TH level
Carlsson et al. 2004 [23]	CHD	37	78 ± 1	55%	20 / 17	folic acid, B6, B12	0.4 mg	10 months	TH level
Doshi et al. 2004 [24]	CHD	100	57 ± 8	88%	50 / 50	folic acid, B6, B12	5 mg	1.5 months	TH level
Hodis et al. 2009 [25]	CHD	506	62 (10)	61%	254/252	folic acid, B6, B12	5 mg	36 months	TH level
Løland et al. 2010 [26]	CHD	174	60 ± 11	84.6%	91 / 83	folic acid, B6, B12	0.8 mg	10.5 months	Restenosis rate
Lonn et al. 2006 [27]	CHD	5522	69 ± 7	71.1%	2758 / 2764	folic acid, B6, B12	2.5 mg	60 months	TH level, Incidence of major vascular disease, Death rate from cardiovascular causes
Schnyder et al. 2001 [28]	Patients undergo angioplasty	205	61 ± 11	80.5%	105 / 100	folic acid, B12	1 mg	6 months	TH level, Restenosis rate, Incidence of major vascular disease
Bleie et al. 2011 [29]	CHD	40	58 ± 9	80%	20 / 20	folic acid, B6, B12	0.8 mg	24 months	TH level

AMI, acute myocardial infarction; NR, no reported; TH, Total homocysteine; CHD, coronary heart disease; FA, folic acid.

The study by Bleie et al. was considered to have potential risk of bias owing to the lack of description regarding the allocation of concealed measures and blind operational procedures [22]. In contrast, the remaining 12 studies employed random allocation methods and provided detailed descriptions of the blinding procedures. All included studies had complete data follow-up and accounted for any losses to follow-up, and clearly described the methods for outcome assessment, and did not exhibit any potential selective reporting. The study by Carlsson et al. was flagged for some concerns regarding potential bias, whereas the others were classified as low risk [23]. Overall, the quality of the included studies was relatively high.

### Meta-analysis

This analysis included 13 RCTs with 14,539 participants (7338 receiving folic acid combined with vitamin B complex and 7301 controls). Eleven studies used folic acid with vitamins B6 and B12, whereas two used folic acid with vitamin B12 only. Table 1 summarizes the studies.

#### TH level

**Figure 2.**
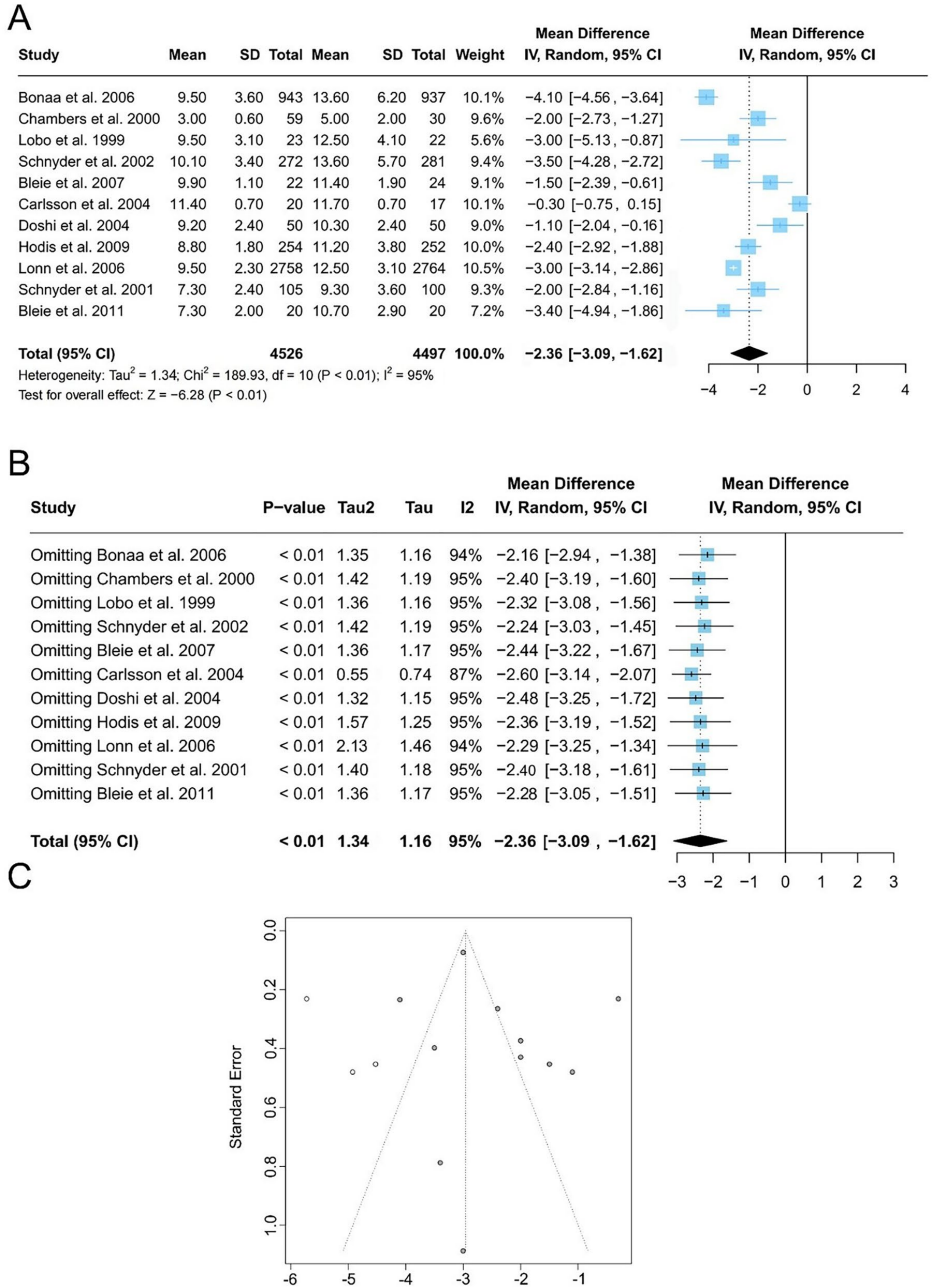
Effect of folate supplementation on serum homocysteine levels between intervention and control groups: **A** forest plot, **B** influence analysis, and **C** funnel plot. SD, standard deviation; Cl, confidence interval; IV, Inverse Variance.

Of the 13 studies included in the analysis, 11 reported the expression of post-intervention TH in serum samples [17–20,22–25,27–29]. Considering the significant heterogeneity across the studies (I^2^ = 95%), a random-effects model was applied, which showed that the expression of TH in the serum was significantly lower in the intervention group than in the control group [MD = −2.36; 95% CI: (−3.09, −1.62); *p* < 0.01] (Figure 2A). A sensitivity analysis was conducted by sequentially excluding each of the 11 studies to assess their influence on heterogeneity and overall effect size. The high heterogeneity persisted regardless of which study was excluded, suggesting that all contributed to the variability and that no single study was a major outlier. Moreover, excluding any individual study did not significantly change the overall effect size, indicating consistency and stability in the findings (Figure 2B). Figure 2C shows the funnel plots for the 11 studies. Egger’s test (*p* = 0.45) and Begg’s test (*p* = 0.34) yielded *p*-values above 0.05, suggesting no significant publication bias or asymmetry in the funnel plot.

#### Restenosis rate

**Figure 3.**
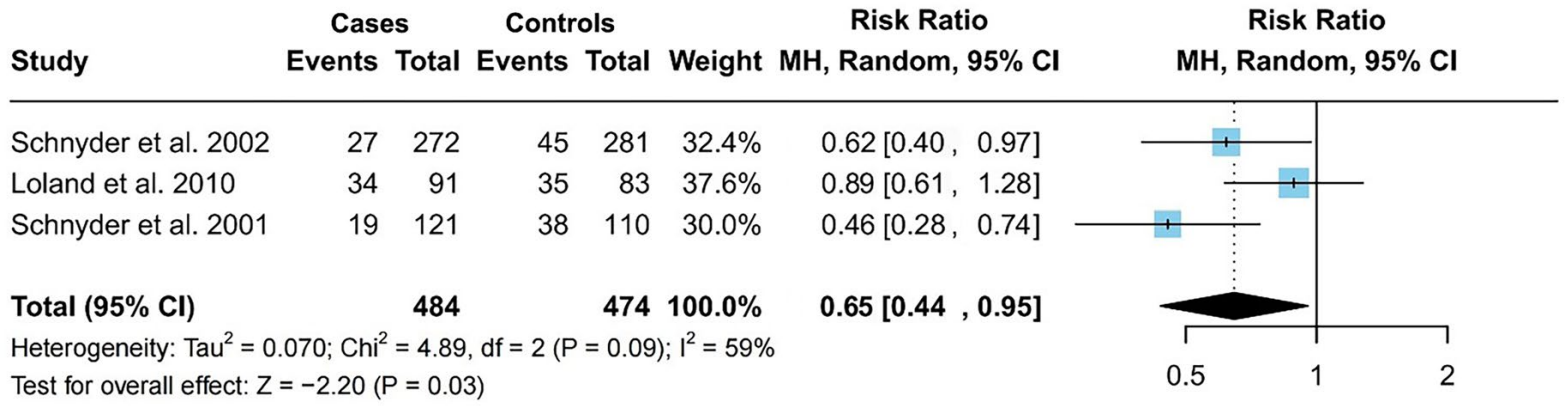
Forest plot of vascular lumen stenosis in the intervention and control groups. Cl, confidence interval.

Among the 13 included studies, three [20,26,28] have documented the incidence of vascular lumen stenosis or restenosis following intervention, revealing significant heterogeneity (I^2^ = 59%). A random-effects model analysis revealed that the incidence of vascular lumen stenosis was significantly lower in the intervention group than in the control group (RR = 0.65; 95% CI: 0.44–0.95; *p* = 0.03; Figure 3).

#### Incidence of major cardiovascular events

**Figure 4.**
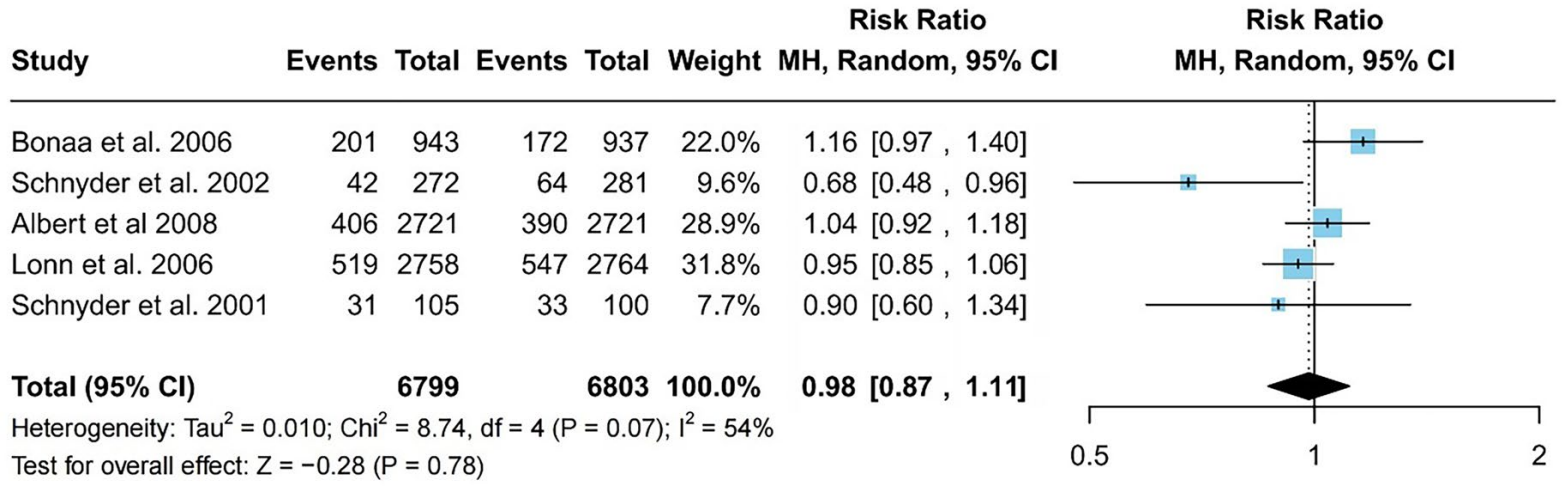
Forest plots of major cardiovascular disease in the intervention and control groups. Cl, confidence interval.

Of the 13 included studies, 5 documented the incidence of major cardiovascular events following intervention, showing notable variability (I^2^ = 54%) [17,20,21,27,28]. A random-effects model analysis revealed no statistically significant difference in the incidence of major cardiovascular event between the intervention and control groups (RR = 0.98; 95% CI: 0.87–1.11; *p* = 0.78; Figure 4).

#### Death rate from cardiovascular causes

**Figure 5.**
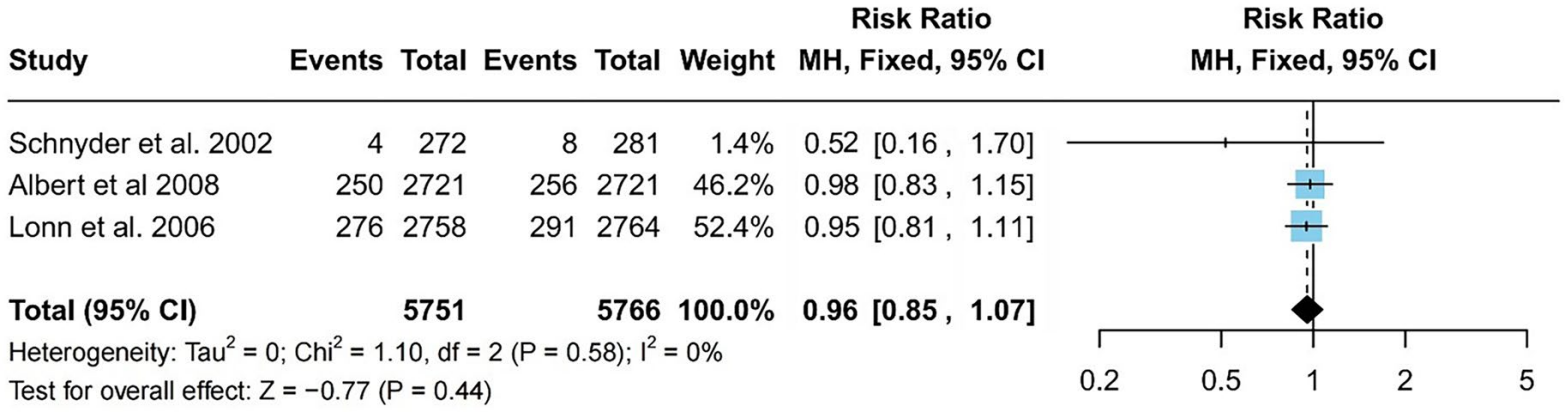
Forest plot of cardiovascular mortality in the intervention and control groups. Cl, confidence interval.

Three of the thirteen included studies reported on the mortality rate owing to cardiovascular causes post-intervention [20,21,27]. With no significant heterogeneity present among the studies (I^2^ = 0%), a fixed-effects model analysis revealed no significant differences in mortality rates between the intervention and control groups (RR = 0.96; 95% CI: 0.85–1.07; *p* = 0.44; Figure 5).

## Discussion

This study suggests that folate supplementation’s cardiovascular disease benefits depend on baseline Hcy levels. This findings is consistent with the results of Miller et al. [30], who reported greater cardiovascular risk reduction in individuals with higher baseline Hcy concentrations. Hcy metabolism relies on two key pathways: the remethylation pathway, which is dependent on folic acid (vitamin B9) and vitamin B12, and the transsulfuration pathway, which requires vitamin B6 [31]. These pathways work in concert to facilitate the effective conversion and elimination of excess Hcy, helping to maintain normal physiological levels. Our findings highlight the synergistic role of folic acid, vitamin B6, and vitamin B12 in achieving optimal regulation of Hcy levels.

Elevated levels of Hcy are well established as an independent risk factor for cardiovascular diseases, particularly CHD [32]. The mechanisms underlying Hcy’s harmful effects are diverse. Elevated Hcy levels can directly damage vascular endothelial cells, leading to impaired endothelial function and increased vascular permeability [33]. This endothelial dysfunction is a key initiating factor in the development of atherosclerosis. Furthermore, Hcy promotes oxidative stress by generating reactive oxygen species, amplifying endothelial injury and inflammation [34]. It also stimulates vascular smooth muscle cell proliferation, contributing to atherosclerotic plaque formation and progression [35]. We found that combined B-vitamin therapy reduced the incidence of long-term vascular lumen stenosis, which aligns with the aforementioned pathological mechanisms. By lowering Hcy levels, the treatment appears to mitigate endothelial injury and slow the progression of atherosclerosis, which is consistent with clinical observations [36]. For instance, a series of coronary angiographies have demonstrated that following successful reduction of Hcy levels, some patients exhibit a decrease in the degree of coronary artery stenosis [37], reinforcing the concept that decreasing Hcy can delay plaque development and improve vascular health [38].

Despite the significant reduction in Hcy levels and vascular stenosis, our study found no significant difference in the incidence of cardiovascular events or mortality between the treatment and control groups. This finding is consistent with those of several other studies that have shown limited clinical benefits of Hcy-lowering therapy on cardiovascular outcomes. For instance, the Vitamin Intervention for Stroke Prevention (VISP) trial reported no significant difference in stroke risk between the high-dose and low-dose B-vitamin supplementation groups [39]. Similarly, the HOPE-2 and VITRO trials did not demonstrate significant reductions in venous thromboembolism events with B-vitamin supplementation [40].

However, some studies have reported conflicting results. For instance, a subgroup analysis of the VISP trial found that high-dose B-vitamin supplementation was linked to an increased risk of ischemic stroke in patients concurrently receiving antiplatelet therapy [41]. This suggests that antiplatelet therapy modifies the effects of B vitamins on cardiovascular outcomes. Furthermore, Shu et al. reported that combined folic acid and vitamin B12 supplementation significantly reduced recurrent deep vein thrombosis (DVT) in patients with cerebral infarction and DVT [42]. These conflicting findings highlight the complexity of the relationship between Hcy levels and cardiovascular events.

Our findings are consistent with the broader literature on Hcy-lowering interventions. A Cochrane review concluded that although B-vitamin supplementation effectively reduces Hcy levels, it does not lead to a significant reduction in cardiovascular events [43]. This suggests that although B vitamins play a key role in regulating Hcy metabolism, their influence on clinical outcomes may be limited by factors such as the use of concomitant medications (e.g. antiplatelet agents) or the multifactorial etiology of cardiovascular disease [44].

Furthermore, the relationship between Hcy and cardiovascular events appears to be more intricate than previously understood. Although elevated Hcy levels are associated with increased cardiovascular risk, the degree to which reducing Hcy levels leads to improved clinical outcomes remains uncertain and widely debated [45]. Although some studies have reported positive effects, others have failed to show significant benefits, underscoring the need for further investigation to clarify the exact role of Hcy in cardiovascular disease and to determine the true effectiveness of Hcy-lowering therapies.

## Conclusions

This study confirmed that combined B-vitamin therapy effectively lowers serum Hcy levels and reduces long-term vascular lumen stenosis but does not alter the incidence of cardiovascular events or mortality. These results carry two key implications. First, they support Hcy reduction as a potential strategy for CHD prevention, informing clinical guidance for high-risk populations with elevated baseline Hcy. Second, the lack of effect on overall cardiovascular events and mortality suggests elevated Hcy is not a central pathogenic driver across all cardiovascular conditions; this helps reconcile conflicting prior studies and narrows the scope of B-vitamin therapy’s potential utility.

While combined B-vitamin supplementation is supported as a targeted CHD preventive measure, its role in broader cardiovascular disease prevention remains uncertain. Future research should focus on identifying specific subgroups (e.g. patients with severe Hcy elevation or early-stage CHD) to further clarify its clinical value.

## Supplementary Material

graphical abstract.png

PRISMA_2020_checklist.docx

Table S1.docx

## Data Availability

All data generated and analyzed during this study are included in this published article and its supplementary information files.
