# Supplementary figures and images for: Combined B-vitamin supplementation on homocysteine and vascular outcomes in coronary heart disease: a meta-analysis
Source: Ann Med. 2026 Jan 30;58(1):2622208. doi: 10.1080/07853890.2026.2622208 (PMC12862861; doi:10.1080/07853890.2026.2622208)

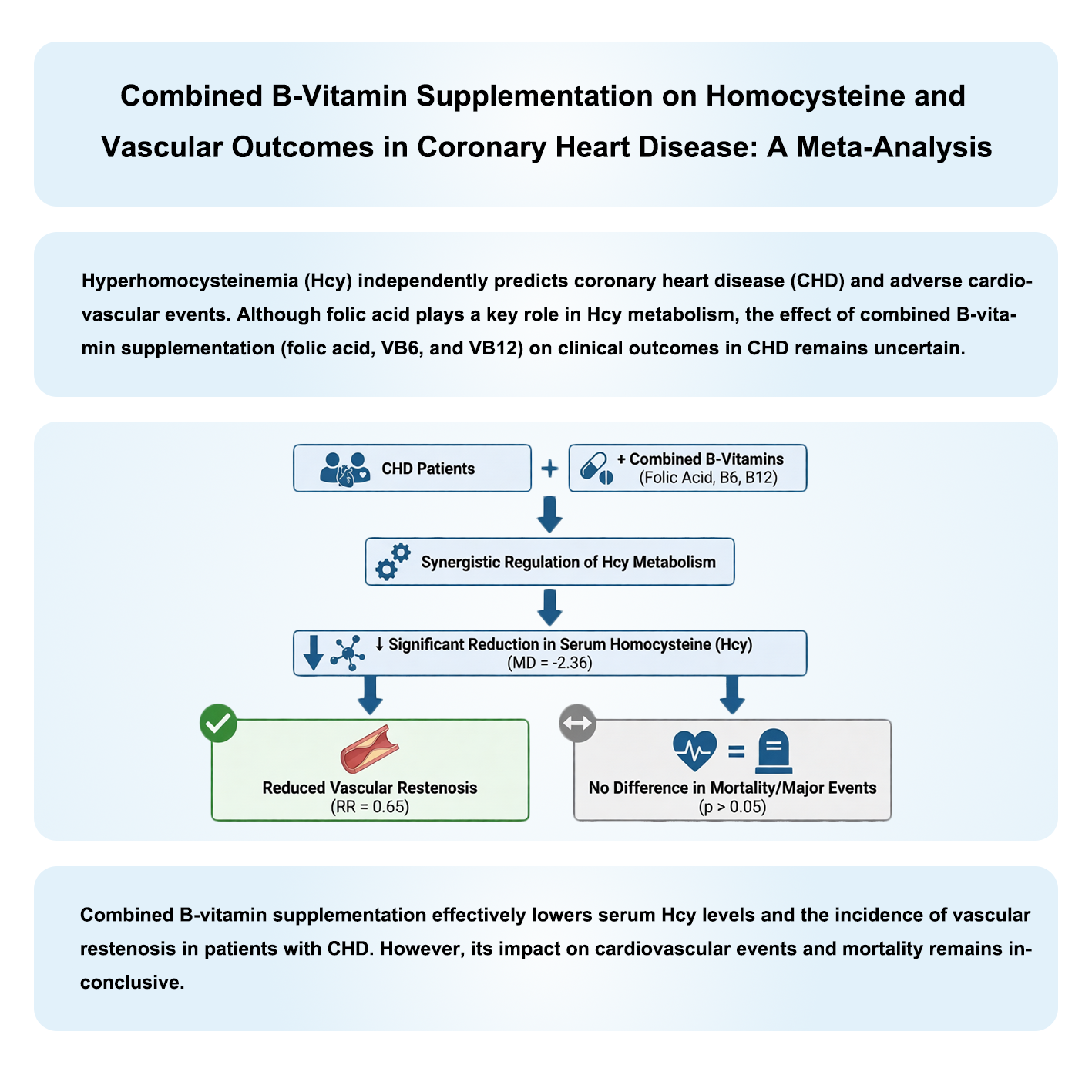

Supplement: graphical abstract.png [file IANN_A_2622208_SM8662.png]
