# Supplementary material for: Combined B-vitamin supplementation on homocysteine and vascular outcomes in coronary heart disease: a meta-analysis
Source: Ann Med. 2026 Jan 30;58(1):2622208. doi: 10.1080/07853890.2026.2622208 (PMC12862861; doi:10.1080/07853890.2026.2622208)
Supplement: Table S1.docx [file IANN_A_2622208_SM8660.docx]

Table S1. Risk of bias in the included studies, assessed by the Cochrane Risk of Bias 2.0 tool

| Study | Randomization | Sample size | Intervention Deviations | Missing Data | Outcome Measurement | Selection of Reported Results | Overall | Weights |
| --- | --- | --- | --- | --- | --- | --- | --- | --- |
| Bønaa et al. 2006 [17] | L | 1880 | L | L | L | L | L | 8 |
| Chambers et al. 2000 [18] | L | 89 | L | L | L | L | L | 8 |
| Lobo et al. 1999 [19] | L | 45 | L | L | L | L | L | 8 |
| Schnyder et al. 2002 [20] | L | 453 | L | L | L | L | L | 8 |
| Albert et al. 2008 [21] | L | 5442 | L | L | L | L | L | 8 |
| Bleie et al. 2007 [22] | L | 46 | L | L | L | L | L | 8 |
| Carlsson et al. 2004 [23] | S | 37 | S | L | L | L | S | 8 |
| Doshi et al. 2004 [24] | L | 100 | L | L | L | L | L | 8 |
| Hodis et al. 2009 [25] | L | 506 | L | L | L | L | L | 8 |
| Løland et al. 2010 [26] | L | 174 | L | L | L | L | L | 8 |
| Lonn et al. 2006 [27] | L | 5522 | L | L | L | L | L | 8 |
| Schnyder et al. 2001 [28] | L | 205 | L | L | L | L | L | 8 |
| Bleie et al. 2011 [29] | L | 40 | L | L | L | L | L | 8 |

H—high risk of bias; L—low risk of bias; S—some concerns; Risk of bias domains: bias arising from the randomization process, bias due to deviations from intended interventions, bias due to missing outcome data, bias in measurement of the outcome, and bias in selection of the reported result.
